# Real-World Evidence of Factors Affecting Cannabidiol Exposure in Children with Drug-Resistant Developmental and Epileptic Encephalopathies

**DOI:** 10.3390/pharmaceutics15082120

**Published:** 2023-08-10

**Authors:** Lucas Brstilo, Gabriela Reyes Valenzuela, Roberto Caraballo, Carlos Pérez Montilla, Facundo García Bournissen, Paulo Cáceres Guido, Paula Schaiquevich

**Affiliations:** 1 Unit of Innovative Treatments, Hospital de Pediatría Prof. Dr. JP Garrahan, Buenos Aires C1245AAM, Argentina; brstilolucas@gmail.com; 2 National Scientific and Technological Research Council (CONICET), Buenos Aires C1033AAJ, Argentina; 3 Neurology Service, Hospital de Pediatría Prof. Dr. JP Garrahan, Buenos Aires C1245AAM, Argentina; gaby_reva@hotmail.com (G.R.V.); robertohcaraballo@gmail.com (R.C.); 4 Multidisciplinary Institute for Research on Pediatric Diseases, Parasitology and Chagas Service, Buenos Aires Children’s Hospital Ricardo Gutierrez, Buenos Aires C1425EFD, Argentina; capm2411@gmail.com; 5 Division of Pediatric Clinical Pharmacology, Department of Pediatrics, Schulich School of Medicine & Dentistry, University of Western Ontario, London, ON N6A 3K7, Canada; facugb1@gmail.com; 6 Pharmacokinetics and Research in Clinical Pharmacology Unit, Hospital de Pediatría Prof. Dr. JP Garrahan, Buenos Aires C1245AAM, Argentina; caceresguido@gmail.com

**Keywords:** cannabidiol, drug-resistant developmental and epileptic encephalopathies, food effect, ketogenic diet, levothyroxine

## Abstract

The identification of factors that affect cannabidiol (CBD) systemic exposure may aid in optimizing treatment efficacy and safety in clinical practice. In this study, we aimed to correlate CBD plasma concentrations at a steady state to demographic, clinical, and pharmacological characteristics as well as seizure frequency after the administration of a purified CBD oil solution in a real-world setting of children with drug-resistant developmental and epileptic encephalopathies (DEEs). Patients receiving oral CBD pharmaceutical products at maintenance were enrolled. Venous blood samples were drawn before the CBD morning dose, 12 h apart from the last evening dose (C0 or CBD trough concentration). A linear mixed-effect analysis was implemented to assess the correlation between C0 and clinical, laboratory, pharmacological, and lifestyle factors. Fifteen females and seven males with a median age of 12.8 years (ranging between 4.7 and 17.2) were included. The median CBD dose was 8.8 mg/kg/day (ranging between 2.6 and 22.5), and the CBD C0 median (range) was 48.2 ng/mL (3.5–366.3). The multivariate model showed a 109.6% increase in CBD C0 in patients with concomitant levothyroxine (β = 0.74 ± 0.1649, *p* < 0.001), 56.8% with food (β = 0.45 ± 0.1550, *p* < 0.01), and 116.0% after intake of a ketogenic diet (β = 0.77 ± 0.3141, *p* < 0.05). All patients included were responders without evidence of an association between C0 and response status. In children with DEEs, systemic concentrations of CBD may be significantly increased when co-administered with levothyroxine, food, or a ketogenic diet.

## 1. Introduction

Cannabidiol (CBD) approval as an add-on therapy for Lennox Gastaut (LGS), Dravet syndrome (DS), and tuberous sclerosis complex in the US and European market has become an effective drug alternative for the treatment of seizures in children with these syndromes. Moreover, recent studies lend support to its use in other less frequent treatment-resistant epilepsy syndromes of different etiologies [1,2].

Despite progress in the field, there are remaining questions that need to be addressed to ensure consistent efficacy among patients and avoid toxicities, including emerging evidence on the pharmacological effect of related components in CBD-enriched oil extracts that may modulate cannabinoid activity [3,4,5,6]. Moreover, the amount and type of excipients in the different pharmaceutical CBD formulations available in the market, the effects of the ketogenic diet, and the type of food and timing of meals in relation to cannabis administration may impact the absorption and systemic exposure to CBD, potentially affecting treatment efficacy and safety [7,8,9,10,11,12]. Part of the variability in CBD systemic exposure may be explained by its lipophilic nature, increased solubility in the presence of high-fat content meals or fatty acids of oil excipients, and increased absorption through the intestinal lymphatic transport process [11,12,13,14]. Other sources of variability in CBD pharmacokinetics include the extensive first-pass liver metabolism after oral administration and pharmacokinetic drug–drug interactions (e.g., by inhibition of cytochrome P450 CYP2C19 and CYP3A4 isoenzymes) [12,13,14,15,16,17]. To our knowledge, the effect of food, diet, and concomitant medication on CBD disposition in children with drug-resistant developmental and epileptic encephalopathies (DEEs) has not been specifically evaluated in real-world cohorts.

In addition, controversial data have been published on the relationship between CBD exposure and seizure control in children to justify therapeutic drug monitoring of CBD so as to target a therapeutic window [18,19].

In this study, we aimed to quantitate CBD trough concentration (CBD pre-dose concentration collected 12 h after CBD dose, C0) at a steady state and study the relation with demographic, clinical, and pharmacological characteristics after the administration of the first oral purified CBD oil solution approved by the national regulatory authority of Argentina in a real-world cohort of children with DEE. Secondary objectives included the assessment of the correlation between CBD C0 and seizure control and short-term tolerability and safety.

## 2. Materials and Methods

The study was approved by the Institutional Review Board at Hospital de Pediatria JP Garrahan (protocol #1046) and was conducted in accordance with the International Conference on Harmonization Good Clinical Practice guidelines and the Declaration of Helsinki. Written informed consent was obtained from parents or caregivers. Pediatric patients with DEEs resistant to standard antiepileptic drugs (AEDs), as well as ketogenic diet therapies, respective epilepsy surgery, and vagus nerve stimulation, who were receiving oral administration of pharmaceutical products containing CBD were enrolled between September 2021 and May 2022.

Pediatric patients aged 2–18 years diagnosed with drug-resistant epilepsy according to the ILAE criteria were included in the study [20]. Enrolled patients were receiving an oral formulation of purified CBD in sesame oil twice daily (Convupidiol^®^, CBD 100 mg/mL, Alef Medical, Buenos Aires, Argentina) for at least 21 days and were on stable doses for longer than one week before C0 collection. The initial dose was 2 mg/kg/day and was up-titrated according to individual tolerability and neurological requirements up to a maximum dose of 25 mg/kg/day. Exclusion criteria included patients who were receiving artisanal cannabis products, intolerance to CBD products, or CBD dose omission the night before the blood sample for C0 assessment was collected. Follow-up visits for routine clinical control and seizure assessment were performed on a twice-monthly basis during the first 6-month period of starting CBD treatment and every 3 months thereafter or at discretion of the treating neurologist.

Drug concentrations and routine laboratory tests in ambulatory patients were obtained after an overnight fast. A peripheral blood sample (2 mL) was collected just before the administration of the morning dose of CBD (steady state trough CBD concentrations or C0). Plasma was separated by centrifugation and stored at −80 °C until assay. Quantification of CBD was performed by means of a validated method using ultrahigh-performance liquid chromatography coupled with tandem mass spectrometry (UHPLC-MS/MS) as previously reported. The assay was linear in the range of 1–300 ng/mL, the lower limit of quantification for CBD was 0.9 ng/mL, and intra-day precision was >96% [21].

Parents or caregivers were interviewed the same day of sample collection to record the following variables: dose (mg) and time of CBD consumption the night before sample collection, days with current CBD dose, time and composition of the dinner provided to the patient the night before, and concomitant medications and/or herbal medicines. CBD dose administration was considered in fed state conditions when the dose was administered between one hour before or after food intake.

### 2.1. Effectiveness

Effectiveness was assessed as the percentage reduction in monthly seizure frequency for each patient. Seizure frequency was calculated at baseline as the number of all seizures averaged over a four-week period before starting CBD treatment according to a standardized seizure diary. Thereafter, percentage reduction in seizure frequency with respect to the baseline value was computed at each visit, with particular attention given to those visits when plasma samples were collected for CBD quantification. Only patients whose seizure frequency decreased by more than 50% (considered as responders) were included in this study [22]. Non-responders or patients who presented serious adverse reactions discontinued CBD treatment. Then, patients were stratified according to the percentage reduction in monthly seizure frequency in two groups: those that showed a percentage reduction between 50% and 74% and the others that were between 75% and 99%, as well as CBD C0 values, were compared between groups [22].

### 2.2. Safety and Tolerability

Routine laboratory analyses and CBD safety assessments were performed at every follow-up visit and as requested based on clinical criteria by the treating neurologist. Concomitant AEDs and dose modifications were recorded during the entire follow-up period.

Adverse effects were recorded by parents in the standardized seizure diary, and neurologists, along with clinical pharmacists, performed an evaluation by recording the incidence, severity, and causality of adverse events using Naranjo’s algorithm [23]. No validated adverse-effect questionnaire was employed.

### 2.3. Statistical Analysis

The power model was used to determine dose proportionality in terms of CBD C0 according to the following equation: C0 = α ⋅ D^β^, where C0 is CBD trough concentration measured for a patient receiving a dose D, and β is the exponent used for examining the proportionality [24].

A linear mixed-effect analysis to assess the relation between CBD C0 and covariates was performed with the package lmer implemented in R (v4.0.4). C0s were log-transformed, and normality assumptions were tested with visual graphical inspection (skewness, kurtosis, histograms, and normal Q-Q plots) and Shapiro–Wilk normality test. Different structural models with an unstructured covariance were tested (random intercept or slope with and without intermodal correlation). The grouping factor was subject ID with random intercept.

A univariate linear mixed effects analysis was performed to identify variables associated with lnC0 that may explain variability among patients in the parameter. A Pearson correlation coefficient was used to study linear associations between lnC0 and continuous variables (age, body mass index, height, and body surface area), while for categorical ones, the effect was considered as one in cases of the concomitant administration with food, ketogenic diet or other drugs (AEDs or other co-administered medicinal products), feeding by a nasogastric tube, and being male (reference sex female = 0). Variables achieving a probable *p*-value < 0.2 in the univariate analysis were included in the multivariate analysis. Then, the final model was obtained after a backward stepwise process at a significance level of *p* < 0.05 and based on the Akaike information criterion (AIC). Linearity, absence of colinearity, homoscedasticity, normality of residuals, and independence of data were assessed in the final model.

CBD C0 values were compared between groups of responders by medians of the Mann–Whitney test. Significance was set at *p* < 0.05. Analyses were carried out by GraphPad Prism version 8.0.1 for Windows, GraphPad Software, San Diego, CA, USA.

## 3. Results

A total of 23 patients were enrolled in the study. The most frequent diagnosis was LGS, followed by other less frequent syndromes, as described in Table 1. One patient was excluded because of the omission of the CBD dose the night before the study. Thus, we finally included 22 patients in the analysis, who had a median age of 12.8 years (ranging between 4.7 and 17.2), comprising 7 males and 15 females. Patient demographics and clinical characteristics are summarized in Table 1 (individual patient characteristics are fully described in Supplementary Materials, Table S1).

Patients were receiving a mean of four AEDs (range, 2–7), and 68% of the cases were treated with four or more AEDs, denoting the severity of the studied cohort. The most frequent AEDs used were levetiracetam (n = 20, 90.9%), sulthiame (n = 12, 54.5%), clobazam (n = 11, 50.0%), and topiramate (n = 12, 40.1%). Of note, seven (31.8%) of our patients were receiving levothyroxine for thyroid dysfunction. Other antiepileptic treatments included vagal stimulation in five patients. Moreover, a ketogenic diet was indicated in five cases, including one patient receiving the classic 4:1 fat-to-protein ratio and the other four with a fat-to-protein ratio + medium-chain triglycerides of 2.1 + 25%, 4.1 + 35%, 3.1 + 5%, and 4.1 + 30%, respectively.

All enrolled patients received CBD orally except for four cases who received the oil solution through a nasogastric tube due to swallowing difficulties. Importantly, almost half of the studied population (n = 10) took CBD with food. As expected for pediatrics, in half of the patients (n = 5), meals were composed only of milk.

One plasma sample was collected from each of the 22 patients except in three cases in which two samples were obtained on different occasions when laboratory tests were performed simultaneously. Therefore, 25 plasma samples corresponding to 22 patients were included in the analysis.

The median CBD dose-normalized C0 was 13.2 (ng/mL)/(mg/kg) with an ample range of 0.8 to 48.5 (ng/mL)/(mg/kg), depicting the high inter-individual variability in the parameter (% coefficient of variation, %CV: 78.8%). Of note, none of the samples had detectable levels of tetrahydrocannabinol in accordance with the lack of this component in the pharmaceutical form.

In the studied dose range, CBD C0 was dose-proportional to the increasing doses of the CBD purified oil as the estimated proportionally coefficient (β) was 1.15 (95%CI: 0.70–1.61, *p* < 0.001). Thus, an increase of 1 mg/kg in CBD dose is expected to result in an increase in CBD C0 of approximately 15% if administering a dose in the studied range.

**Figure 1:**
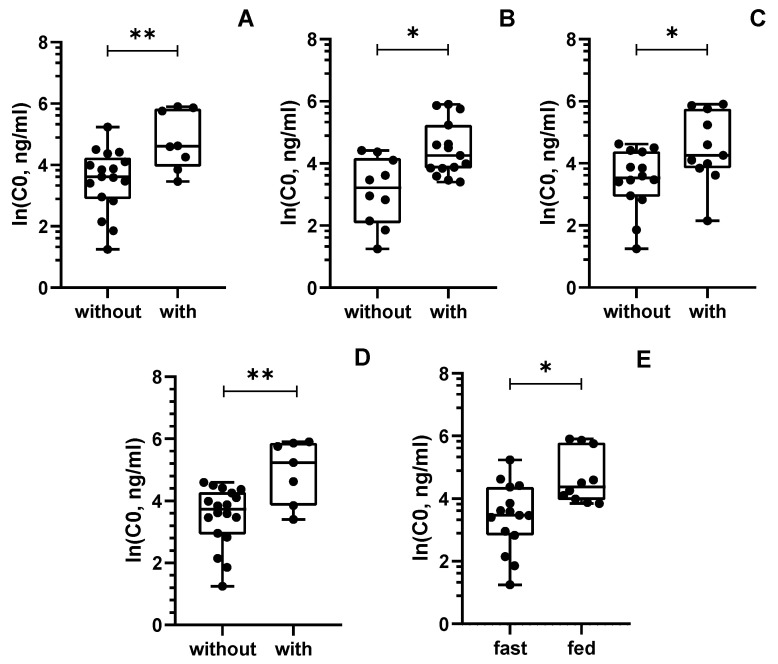
Relations between cannabidiol C0 and concomitant administration of (**A**) levothyroxine, (**B**) sulthiame, (**C**) clobazam, (**D**) ketogenic diet, and (**E**) food. CBD trough concentrations are represented in the natural logarithmic scale. * *p* < 0.05; ** *p* < 0.01.

A linear mixed effects analysis was conducted to uncover significantly related factors to CBD C0. Firstly, CBD C0 values were log-transformed to follow a normal distribution. On univariate analysis, the results showed significant positive associations between ln(C0) and concurrent usage of levothyroxine, sulthiame, or clobazam, whereas the ketogenic diet therapy or concomitant food administration were also notably associated with ln(C0) (Table 2). Patients who were concomitantly receiving levothyroxine presented a median CBD C0 of 100.7 ng/mL vs. 37.3 ng/mL observed in those free of the thyroid hormone (Figure 1A, *p* < 0.01). Moreover, patients receiving sulthiame also had a higher median CBD C0 than those without this AED (median, 53.9 ng/mL vs. 19.2 ng/mL, respectively) (Figure 1B, *p* < 0.05). Similarly, children receiving clobazam showed higher CBD C0 (median, 70.6 ng/mL vs. 32.0 ng/mL) (Figure 1C, *p* < 0.05). Notably, CBD C0 values in patients under ketogenic diet therapy were significantly higher than those obtained under regular diets (median, 101.9 ng/mL vs. 36.8 ng/mL, respectively) (Figure 1D, *p* < 0.01), while children who received CBD in fed conditions also presented significantly higher CBD C0 than those who received it in fast conditions (median, 70.0 ng/mL vs. 34.1 ng/mL, respectively) (Figure 1E, *p* < 0.05).

After backward elimination, the final multivariate model retained levothyroxine co-administration (β = 0.74 ± 0.1649, *p* < 0.001), ketogenic diet (β = 0.77 ± 0.3141, *p* < 0.05), and fed state (β = 0.45 ± 0.1550, *p* < 0.05), as summarized in Table 3. Thus, patients receiving levothyroxine treatment, those who were on a ketogenic diet, or those who took CBD in fed condition had a 109.6% (95% CI, 49.2–194.5), 116.0% (95% CI, 12.7–313.7), and 56.8% (95% CI, 13.9–116.0) increase in CBD C0, respectively. According to this model, the CBD C0 level of a hypothetical patient undergoing levothyroxine therapy, adhering to a ketogenic diet, and taking CBD with food would be expected to be 2.8-fold higher than that of children without hormone treatment, ingesting a regular diet, and in a fasted state.

### 3.1. CBD Plasma Levels in Relation to Seizure Control

**Figure 2:**
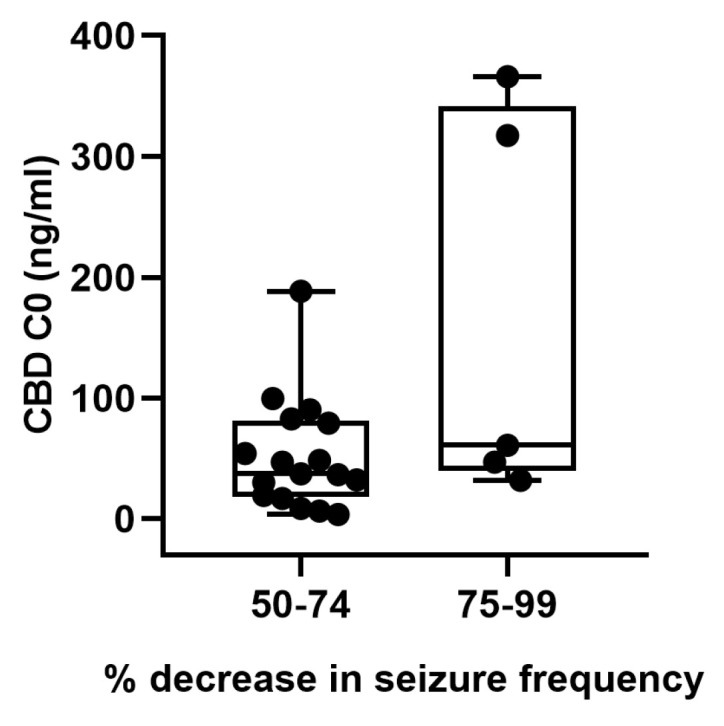
Relationship between the percentage decrease in seizure frequency and cannabidiol trough concentrations.

To note, all patients included in the present study were responders, of whom 17 (77.3%) had a 50–74% decrease in seizure frequency, and 5 (22.7%) had a decrease in the parameter between 75 and 99%. The clinical characteristics of both groups are described in Supplementary Material (Table S2). We detected no significant differences in median CBD C0 between patients who had a decrease in seizure frequency of 50–74% and those with a 75–99% change (42.1 ng/mL vs. 53.7 ng/mL, respectively) (Figure 2, *p* > 0.05). In correspondence, no statistical differences were observed in CBD daily doses between the two groups of responders who showed a median (range) CBD daily dose of 8.3 mg/kg/day (2.6–22.5) and 8.5 mg/kg/day (3.7–15.3) in cases that achieved a 50–74% and 75–99% decrease in seizure frequency, respectively (*p* > 0.05). 

### 3.2. Safety and Tolerability

Overall, the CBD oral solution was well tolerated. Routine laboratory tests showed no signs of liver dysfunction in any patient during the follow-up period. Only three adverse events in 22 patients were registered at 32, 43, and 94 days after starting CBD. All were mild, including two cases of somnolence in patients receiving 4 mg/kg/day and 10 mg/kg/day of CBD and one decrease in appetite that developed at CBD 12 mg/kg/day. Somnolence was resolved in both cases after lowering the clobazam dose by 25%, and appetite improved by lowering the dose of CBD to 10 mg/kg/day. According to Naranjo’s algorithm, the three adverse events were classified as possible.

Importantly, no patient discontinued CBD due to drug-related adverse events.

## 4. Discussion

The advent of CBD use as an add-on therapy has become an effective drug for the treatment of children with drug-resistant DEE worldwide. Nonetheless, the widespread use of CBD, especially beyond the better-studied indications such as LGS and DS, requires further clinical and pharmacological studies to aid healthcare professionals in patient management. In this study, we present novel findings on the impact of food, diet therapy, and concomitant medications on CBD trough plasma concentrations. We observed a significant increase in CBD C0 in patients who received the cannabinoid with concomitant levothyroxine treatment, in those who were on a ketogenic diet, and in patients who took the CBD oil solution with their evening meal.

In our cohort, the median daily dose of CBD was 8.5 mg/kg/day, which was lower than the doses reported in open-label studies in pediatrics, which ranged between 14.5 and 22.0 mg/kg/day [25,26,27]. These differences may be related to changes in pharmaceutical excipients that modify CBD bioavailability and, therefore, the efficacy of the cannabinoid or due to the differences in the composition of medicinal drug products that contain different pharmacologically active components that may act synergistically, reducing the required dose for seizure control among other factors [2,3,11,13].

The relation between CBD daily dose and CBD C0 concentration was approximately proportional in the studied CBD dosing range (2.6 to 22.5 mg/kg/day). This result is in accordance with studies that reported a linear relationship between CBD dose and CBD plasma concentrations in similar and even higher dose ranges (i.e., 5 to 40 mg/kg/day) [18,19,28,29].

As expected, we observed a remarkable variability in CBD dose-normalized C0 among our patients (%CV: 76.5%). Sources of inter-patient variability in CBD C0 may include the lipophilic nature of the drug that contributes to the variable and erratic absorption of the drug, differences in oil vehicle composition of CBD formulations, lifestyle factors (e.g., consumption of CBD with food), concomitant administration of drugs that alter the activity of cannabidiol metabolizing enzymes, the route of drug administration (oral swallowing versus administration through a nasogastric tube), age-related gastrointestinal developments, and possibly uncertainty on exact dosing times. Despite the high variability observed, the median CBD dose-normalized C0 (C0/D) in our cohort was similar to other studies in children [19,28,29,30].

Administration of CBD along with high-fat/calorie meals has been shown to increase systemic exposure by four-fold compared to the fasted state in adult healthy volunteers and patients [7,8,9,10,11]. However, it is worth noting that no evidence is currently available on the pharmacokinetic effects of meals on CBD in children. Thus, due to the lack of sufficient evidence in children, there are no recommendations to guide CBD dosing that take into account the potential effects of food and diet. Specifically, food intake prolongs gastric transit time and may result in higher fractions of the drug becoming capable of being dissolved in gastrointestinal fluids [31,32]. Moreover, the content is particularly important as a high-fat meal stimulates bile secretion, which improves CBD solubilization and, thereafter, absorption of this lipophilic drug. In agreement with the reports in adults, we detected a 57% increase in CBD C0 in the fed state of our patients. Nonetheless, the magnitude of the food effect on CBD systemic exposure was much lower in our pediatric cohort, probably because more than half of our patients received only milk in contrast to high-fat meals administered in the abovementioned adult studies.

Even though few studies assessed possible interactions between the ketogenic diet and serum concentrations of AEDs, there are no previous reports that evaluated the influence of this diet therapy on CBD plasma concentrations [33,34]. For the first time, we report that patients who were receiving a ketogenic diet showed an approximately 115% increment in CBD plasma concentrations compared to children on regular diets. The ketogenic diet is composed of high-fat meals, potentially increasing CBD absorption and bioavailability due to the lipophilic nature of the cannabinoid. Zgair et al. (2016) suggested that the primary mechanism of the increased absorption of cannabinoids in the presence of lipids is intestinal lymphatic transport [35]. Thus, drugs that are transported via the intestinal lymphatic system avoid hepatic first-pass metabolism and therefore achieve higher bioavailability. Hence, the elevated amount of lipids and triglycerides in the ketogenic diet may enhance the emulsification and micellar solubilization of CBD and activate lymphatic transport, which in turn may increase its intestinal absorption, avoid first-pass metabolism and, thus, elevate systematic exposure [36,37,38]. Still, this hypothesis should be validated by appropriate mechanistic models.

CBD is subject to remarkable liver first-pass metabolism mostly by cytochrome P450 isoforms CYP2C19, CYP3A4, and CYP2C9, as well as phase II enzymes that mediate glucuronidation (UGT1A7, UGT1A9, and UGT2B7) [15,16]. Moreover, CBD inhibits, at least in vitro, several CYP450 isoforms that constitute the main biotransformation pathways of the most common AEDs [15,16,39,40,41]. It is thus possible that polymedicated patients, such as children with drug-resistant DEE, could be at risk of developing drug–drug interactions with CBD. In accordance with our previous studies in patients treated with an enriched CBD oil formulation, we observed notably higher CBD C0 in levothyroxine-treated patients [24]. Experimental results from in vitro and animal studies strongly suggest that thyroid hormones reduce CYP3A activity in humans, affecting the main pathway for CBD metabolization [42]. Also, levothyroxine-mediated UGT inhibition has been described [43]. Both findings in the present study and our previous report are the only evidence of the influence of levothyroxine on CBD exposure. Most probably, we have been able to determine this relationship due to the high proportion of epileptic patients treated for hypothyroidism in our hospital with traditional anticonvulsants such as valproic acid and carbamazepine with precedents of thyroid hormone metabolic disturbances [44,45].

Regarding concomitant AEDs, clobazam and sulthiame were significantly associated with CBD C0 in the univariate analysis but were not retained in our final model. Among CBD-drug interactions, studies in adults and children have shown pharmacokinetic interactions between CBD and clobazam as a consequence of the inhibition of the biotransformation of the active metabolite norclobazam mediated by CYP3A4 and CYP2C19 [25,26,27,28,29,41,46,47,48].

Due to the frequent use of clobazam in DEEs, it is important to note that CBD C0s were 100% higher in patients receiving benzodiazepine compared to those free of it. This result is in line with other trials in which a bidirectional interaction between CBD and clobazam has been reported [28,40,49]. The underlying mechanism of this interaction might be explained by the main metabolite of clobazam, norclobazam, which inhibits CBD UGT-mediated metabolism of CBD [40]. Nonetheless, clobazam concomitant administration was not retained in the final model. Some possible explanations are related to the CYP2C19 genotype, as norclobazam plasma levels are five-fold lower in extensive metabolizers than in poor metabolizers, leading to unaltered CBD UGT-mediated metabolism in extensive metabolizers patients. Moreover, norclobazam increased exposure may not occur in the presence of other CYP2C19 inhibitors, such as sulthiame, as previously reported for stiripentol [29]. Also, the knowledge in advance of the interaction may have prompted the neurologists to decrease clobazam doses at the first symptom of somnolence and sedation.

In our clinical center, sulthiame is prescribed in children with refractory epilepsy due to its proven efficacy in LGS [50]. We observed significantly higher CBD C0 in patients concomitantly receiving sulthiame as part of the AED treatment, hypothetically explained by the inhibition of CYP2C19-mediated metabolism of CBD [51]. Anecdotal use of sulthiame in large clinical centers with vast CBD experience may have hindered previous detection of this drug–drug interaction. This highlights the importance of conducting further pharmacokinetic studies in larger and more diverse pediatric patients with DEEs.

We found no correlation between seizure control and CBD doses within the range studied, consistent with other research [19,52,53]. In addition and in accordance with previous studies in children, we failed to show a relation between CBD exposure measured as trough concentrations and response status [19,54]. This finding may be related to the fact that CBD is a highly fat-soluble substance, and plasma concentrations may not exactly match concentrations in the brain. Despite the documented penetration of CBD into the brain of animals, the presence of the blood–brain barrier hinders the free transport into the tissue [55,56]. Moreover, it is well described that various anatomical regions of the brain present diverse physiological activity due to their differences in structure, and therefore, differences in CBD concentrations may occur within the brain that may impact seizure control [56,57]. Nonetheless, further studies should be carried out to dive into CBD pharmacokinetic-pharmacodynamic relations to potentially support therapeutic drug monitoring of CBD for routine patient management.

The CBD oral solution was safe and very well tolerated in our patients after one year of follow-up. Compared to previous reports, somnolence and decreased appetite were expected, as these are one of the most frequent CBD-associated adverse events reported in pediatric and adult patients [25,26,27,38,44,45,46]. The low incidence of adverse drug reactions may be explained by the lower median dose compared to other studies and because it was a study cohort under long-term CBD treatment, since the onset of most adverse events is more probable during the first month of treatment.

The retrospective nature of this study and the small sample size of our cohort limits the statistical power of the analysis. This is a real-world study, and therefore, the exact time that CBD was administered the night before blood collection could not be ascertained to exactly calculate the time post-dose that the blood sample for CBD quantification was obtained. Also, the exact time, duration, and fat/calorie composition of the meal the patient consumed were recorded according to what the parents reported, but deviations could have occurred, affecting the residual variability of the analysis. We also should acknowledge that the results of this study using an oral pharmaceutical-grade cannabidiol oil-based solution may not be generalizable to other products; however, it does provide a means to account for the whole dose and gives results under optimal conditions. Despite the limitations, the present results also reflect the value of using real-world data obtained from a pediatric outpatient at the largest clinical center in our country and the impact of the results on clinical management and patient counseling.

## 5. Conclusions

The most relevant findings were the evidence of a significant increase in CBD C0 when administered with levothyroxine as comedication, in patients with a ketogenic diet, and when administered concomitantly with food. These findings could potentially assist the medical staff in managing patients who are being treated with CBD.

## Figures and Tables

**Table 1:** Demographic, clinical, and pharmacological characteristics.

Characteristic/Parameter	Results ^a^
Age, years	12.8 (4.7–17.2)
Sex, n	Female, 15 (68.2%); male: 7(31.8%)
Weight, kg	35.0 (11.4–70.0)
Height, cm	140.0 (104.0–158.0)
Epileptic syndrome (number of patients)	LGS (11), MAE (Doose syndrome) (5), CSWSS (2), WS (2), frontal epilepsy (1), and myoclonic epilepsy (1)
Concomitant drugs (number of patients)	Levetiracetam (20), Sulthiame (12), Clobazam (11), Topiramate (9), Levothyroxine (7), Zonisamide (7), Rufinamide (5), Valproic acid (5), Baclofen (4), Diazepam (4), Ethosuximide (3), Lamotrigine (2), Lorazepam (2), Lacosamide (2), Levomepromazine (2), Risperidone (2), Omeprazole (2), and Clonazepam (1)
CBD dose, mg/kg/day	8.5 (2.6–22.5)
CBD C0, ng/mL	48.2 (3.5–366.3)
CBD C0/D, (ng/mL)/(mg/kg/day)	6.8 (0.4–24.2)
CBD duration treatment (days)	749 (43–1224)

Abbreviations: C0: cannabidiol trough concentration; CBD: cannabidiol; CBD C0/D: dose-normalized cannabidiol trough concentration; CSWSS: continuous spikes and waves during slow sleep; D: dose; LGS: Lennox Gastaut syndrome; MAE: myoclonic-atonic epilepsy; and WS: West syndrome. ^a^ Numerical continuous data are expressed as median (range).

**Table 2:** Univariate linear mixed effect analysis of variables associated with CBD trough concentrations.

Variable	Estimate (β)	Standard Error	*p*-Value
Levothyroxine	1.40	0.2485	<0.01
Sulthiame	1.42	0.2598	0.02
Clobazam	1.42	0.3591	0.03
Ketogenic diet	1.51	0.2443	<0.01
Food administration	1.34	0.5274	0.02

Levothyroxine, sulthiame, and clobazam median (range) dose was 50.0 µg/day (37.5–100.0), 400 mg/day (200–600), and 20 mg/day (10–30), respectively. In the model, ketogenic diet or food administration took the value of 1 or 0 if the patient was co-treated with ketogenic diet or whether or not food was provided concomitantly to CBD (one hour previous or after CBD administration), respectively.

**Table 3:** Multivariate linear mixed effect analysis of variables associated with CBD trough concentrations.

Variable	Estimate (β)	Standard Error	*p*-Value
Intercept	−2.42	1.0779	0.03
lnD	1.15	0.2201	<0.001
Levothyroxine	0.74	0.1649	<0.001
Food administration	0.45	0.1550	0.009
Ketogenic diet	0.77	0.3141	0.02

The final model is described by the following equation: ln(C0) = −2.42 + 1.15 × ln (D) + 0.74 × levothyroxine + 0.45 × food administration + 0.77 × ketogenic diet + (1|ID), in which ID means subject and D means dose. Levothyroxine, food administration, and ketogenic diet can take the value of 1 or 0 if the patient was co-treated with the hormone thyroid, if the drug or food was provided concomitantly to CBD, and if the patient was under ketogenic diet treatment or not, respectively.

## Data Availability

The data that support the findings of this study are in the article or as supplementary material.

## Supplementary Materials

The following supporting information can be downloaded at https://www.mdpi.com/article/10.3390/pharmaceutics15082120/s1. Table S1: Full description of the enrolled patients; Table S2: Clinical, demographic, and pharmacokinetic characteristics in relation to seizure control.

## Abbreviations

AEDs	antiepileptic drugs
C0	trough concentration
CBD	cannabidiol
CSWSS	continuous spikes and waves during slow sleep
D	dose
DS	Dravet syndrome
DEE	developmental and epileptic encephalopathies
LGS	Lennox Gastaut syndrome
Ln	natural logarithmic
MAE	myoclonic-atonic epilepsy
WS	West syndrome
