# Supplementary material for: Real-World Evidence of Factors Affecting Cannabidiol Exposure in Children with Drug-Resistant Developmental and Epileptic Encephalopathies
Source: Pharmaceutics. 2023 Aug 10;15(8):2120. doi: 10.3390/pharmaceutics15082120 (PMC10459683; doi:10.3390/pharmaceutics15082120)
Supplement: Supplementary file 1 [file pharmaceutics-15-02120-s001.zip › Brstilo_Table S1.pdf]

Table S1. Full description of the enrolled patients

ID	Sex/ Age, years	Ht, cm/ Wt, kg	Epileptic syndrome	Concomitant drugs (dose, mg/day)	CBD dose, mg/kg/day	CBD C0, ng/ml [a]	Other treatment
**1**	F/13.4	140/33.2	LGS	LEV (2500), ZNS (100), TPM (200), SLT (300)	10.5	70.6	VNS
**1**	F/13.8	140/34.0	LGS	T4 (37.5)*, LEV (2500), ZNS (100), TPM (200), SLT (300)	10.3	99.4	VNS
**2**	F/14.9	155/60	CSWSS	T4 (100) *, SLT (400), ETS (500), CLN (0.5)	5.0	31.9	-
**3**	F/11.9	143/38.5	LGS	LEV (3000), ZNS (400), CLB (30), VPA (750)	11.7	188.3	KD
**4**	M/7.8	104/18.2	LGS	LEV (3000), ZNS (100), CLB (20), DZP (10)	19.2	37.3	-
**5**	F/10.1	119/20.6	CSWSS	T4 (37.5) *, SLT (400), CLB (20), ETS (500), DZP (20), BAC (20)	14.6	353.0	KD
**5**	F/10.5	122/19.6	CSWSS	T4 (37.5) *, SLT (400), CLB (20), ETS (500), DZP (20), BAC (20)	15.3	366.3	KD
**6**	M/17.2	155/35.2	LGS	T4 (50) *, LEV (3000), ZNS (100), TPM (50), SLT (600), CLB (30), ETS (500), RUF (800), OMEP (10)	14.2	317.5	VNS
**7**	F/7.8	118/21	WS	LEV (1000), TPM (75)	7.1	19.2	-
**8**	F/13.7	127/38	MAE	LEV (2500), TPM (300), BAC (30), LCM (400), LRZ (2)	4.2	16.9	-
**9**	F/11.2	105/11.4	WS	LEV (1500), ZNS (600), BAC (10), OMEP (10), LEVOM (3.125)	8.8	3.5	-
**10**	M/12.5	135.5/33.6	MAE	LEV (1000), ZNS (100), SLT (200), CLB (30), RUF (400), RISP (1)	8.9	48.2	VNS
**11**	F/13.6	143/70	LGS	LEV (4000), SLT, (600) CLB (20), VPA (500)	7.9	53.9	-
**12**	M/4.7	104.5/20	MAE	LEV (2000), TPM (50), RUF (1400), RISP (4)	22.5	82.7	-

**13**	F/13.4	130/38.5	LGS	LEV (2500), RUF (800), LRZ (6)	2.6	6.4	-
**14**	F/12.1	141/40.5	LGS	LEV (3000), SLT (400), CLB (20), VPA (1500)	3.7	46.7	KD, VNS
**14**	F/12.4	144/42	LGS	T4 (50)*, LEV (3000), SLT (400), CLB (20), VPA (1500)	6.0	101.9	KD, VNS
**15**	F/16.6	152/35	MAE	LEV (3000), SLT (600), VPA (500)	11.4	90.3	-
**16**	F/13.0	147/32.5	LGS	LEV (3000), TPM (350), CLB (25), RUF (600)	4.0	8.6	-
**17**	F/12.2	124.6/50	LGS	T4 (37.5) *, LEV (3000), LTG (100), CLB (20)	11.0	79.1	-
**18**	F/13.1	141/38.6	LGS	LEV (3000), TPM (300), SLT (200), DZP (8), LEVOM (75), LCM (150)	5.2	30.0	KD
**19**	M/14.6	143/32.5	Myoclonic epilepsy	LEV (2500), TPM (400), SLT (400), DZP (7.5), BAC (50)	3.1	36.3	-
**20**	M/7.2	119.5/28.6	LGS	T4 (50)*, LEV (2500), SLT (200), VPA (1250)	5.2	47.0	KD, VNS
**21**	M/12.8	142/53	MAE	LEV (1000), ZNS (200), CLB (20)	8.5	60.7	-
**22**	F/15.6	158/41	Frontal epilepsy	LEV (2000), LTG (200), TPM (300)	4.9	32.2	-

[a]Two C0 values are reported in those cases that patients were studied on two occasions.

*Doses are expressed as µg/day

Abbreviations: BAC, baclofen; C0: cannabidiol trough concentration; CBD: cannabidiol; CLB, clobazam; CSWSS: continuous spikes and waves during slow sleep; D: dose; DZP, diazepam; ETS, ethosuximide; F, female; KD, ketogenic diet; LCM, lacosamide; LEV, levetiracetam; LEVOM, levomepromazine; LGS: Lennox Gastaut syndrome; LRZ, lorazepam; LTG, lamotrigine; MAE: myoclonic-atonic epilepsy; M, male; OMEP, omeprazole; RISP, risperidone; RUF, rufinamide; T4, levothyroxine; SLT, sulthiame; TPM, topiramate; VNS, vagus nerve stimulation; VPA, valproic acid; WS, West syndrome; ZNS, zonisamide
