# Supplementary material for: Real-World Evidence of Factors Affecting Cannabidiol Exposure in Children with Drug-Resistant Developmental and Epileptic Encephalopathies
Source: Pharmaceutics. 2023 Aug 10;15(8):2120. doi: 10.3390/pharmaceutics15082120 (PMC10459683; doi:10.3390/pharmaceutics15082120)
Supplement: Supplementary file 1 [file pharmaceutics-15-02120-s001.zip › Brstilo_Table S2.pdf]

Table S2. Clinical, demographic, and pharmacokinetic characteristics in relation to seizure control

**Characteristic/parameter**	*Percentage decrease in seizure frequency*	
	**50-74%**	**75-99%**
Sex (female/male), n	12/5	3/2
Age, years	13.0 (4.7-16.6)	12.4 (10.1-17.2)
CBD C0, ng/mL	37.3 (3.5-188.3)	60.7 (31.9-366.3)
CBD daily dose, mg/kg/day	7.9 (2.6-22.5)	8.5 (3.7-15.3)
Epileptic syndrome, n (%)		
LGS	9 (53.0)	2 (40)
MAE	4 (23.5)	1 (20)
CSWSS	-	2 (40)
WS	2 (11.7)	-
Myoclonic epilepsy	1 (5.9)	-
Frontal epilepsy	1 (5.9)	
Number of AEDs	4±2	3±2
Other interventions n, (%)		
Ketogenic diet	3 (17.6)	2 (4.0)
Vagus nervous stimulation	3 (17.6)	-

*Continuous data are expressed as median (range)

Abbreviations: AED, antiepileptic drug; C0, cannabidiol trough concentration; CBD, cannabidiol; CSWSS, continuous spikes and waves during slow sleep; LGS, Lennoux-Gastaut syndrome; MAE, myoclonic atonic epilepsy, WS, West syndrome
